# Bayesian inference of population size history from multiple loci

**DOI:** 10.1186/1471-2148-8-289

**Published:** 2008-10-23

**Authors:** Joseph Heled, Alexei J Drummond

**Affiliations:** 1 Department of Computer Science, University of Auckland, Auckland, New Zealand; 2 Bioinformatics Institute, University of Auckland, Auckland, New Zealand

## Abstract

**Background:**

Effective population size (*N*_*e*_) is related to genetic variability and is a basic parameter in many models of population genetics. A number of methods for inferring current and past population sizes from genetic data have been developed since JFC Kingman introduced the n-coalescent in 1982. Here we present the Extended Bayesian Skyline Plot, a non-parametric Bayesian Markov chain Monte Carlo algorithm that extends a previous coalescent-based method in several ways, including the ability to analyze multiple loci.

**Results:**

Through extensive simulations we show the accuracy and limitations of inferring population size as a function of the amount of data, including recovering information about evolutionary bottlenecks. We also analyzed two real data sets to demonstrate the behavior of the new method; a single gene Hepatitis C virus data set sampled from Egypt and a 10 locus *Drosophila ananassae *data set representing 16 different populations.

**Conclusion:**

The results demonstrate the essential role of multiple loci in recovering population size dynamics. Multi-locus data from a small number of individuals can precisely recover past bottlenecks in population size which can not be characterized by analysis of a single locus. We also demonstrate that sequence data quality is important because even moderate levels of sequencing errors result in a considerable decrease in estimation accuracy for realistic levels of population genetic variability.

## Background

Coalescent theory has been described as "the most significant progress in theoretical population genetics in the past two decades of [last] century" [1]. Methods based on coalescent theory enable estimation of both current and past population sizes directly from genetic data. Effective population size (*N*_*e*_) is linked to the rate of genetic drift and inbreeding, and is useful for example when investigating the possibility of interbreeding between Neanderthals and early humans [2], or when looking at patterns of genetic variation in human genes [3]. Even when population size history is of secondary interest the incorporation of models of population size may improve the genetic mapping of diseases and estimation of genetic traits [4].

### A concise introduction to the coalescent

The coalescent was formally introduced by Kingman in 1982 [5]. This was the culmination of eight years of developing the "circle of ideas known as the coalescent" [6] which brought into light the essence of the relation between population size and ancestry. Informally, the larger the population, the longer any two individuals have to trace their ancestry back in time until meeting their common ancestor (*concestor *[7]). The meeting of those two ancestral lineages is known as the *coalescence event*.

To formally develop the theory one has to assume the population is mixing perfectly so that all members of the same generation have equal probability of being the ancestor of any member in the next generation. Kingman introduces the idea using an idealized Wright-Fisher population but shows how the Moran model also has the coalescent as its diffusion limit (for a good summary of these models see [8]).

Consider two random members from a population of fixed size *N*. By perfect mixing, the probability they share a concestor in the previous generation is 1/*N*. The probability the concestor is *g *+ 1 generations back is $\frac{1}{N}{\left(1-\frac{1}{N}\right)}^{g}$. This elementary reasoning shows that *g*, as a random variable, has a geometric distribution with a success rate of *λ *= 1/*N*, and so has mean *N *and variance of *N*^3^/(*N *- 1).

With *n *lineages the time to the first coalescence is derived in the same way, only now there are $\left(\begin{array}{c} n\\ 2 \end{array}\right)$ possible pairs that may coalesce, resulting in a success rate of $\lambda =\left(\begin{array}{c} n\\ 2 \end{array}\right)/N$ and mean time to coalescence of $N/\left(\begin{array}{c} n\\ 2 \end{array}\right)$.

This assumes *N *is much larger than *O*(*n*^2^). An interesting consequence is that the total number of generations required for *n *lineages to coalesce into one is $\sum_{i=2}^{n}N/\left(\begin{array}{c} i\\ 2 \end{array}\right)=2N(1-1/n)$, which is always less than 2*N *regardless of *n*.

Kingman goes on to show that as *N *grows the coalescent process converges to a continuous time Markov chain. For the above *λ *= 1/*N *is the instantaneous probability of coalescing, i.e. the probability of coalescing on a short time interval Δ*t *is *O*(*λ*Δ*t*). Unsurprisingly the solution turns out to be the exponential distribution *f*(*t*) = *λe*^-*λ*t^, the continuous equivalent of the geometric distribution.

While Kingman mentions that population size does not have to be constant the details are not given. The generalization of the coalescent for the case where the population size changes over time, *N *= *N*(*t*) is given by Griffiths and Tavare [9]. They showed that the coalescent density for the first coalescence event being at time *t *in the past given *n *lineages is:

(1) $$\begin{array}{ccc} f(t)=\frac{C}{N(t)}\exp\left(-\int_{0}^{t}\frac{C}{N(x)}dx\right) & \text{where} & C=\left(\begin{array}{c} n\\ 2 \end{array}\right). \end{array}$$

In the rest of the article we shall call *N *= *N*(*t*) the *demographic function *or sometimes just *demographic *when the context is clear. Note that while *N*(*t*) may take any form whose inverse can be integrated, the density is characterized by two numbers only, the intensity (average of population size inverse over the interval) and population size at the end of the interval.

All together, given a demographic function *N*(*t*) and a list of coalescence times *T *= (*t*_*n*+1 _= 0, *t*_*n*_, *t*_*n*-1_,...*t*_2_) where *t*_*n *_is the time *n *lineages has coalesced into *n *- 1, the probability those times are the result of the coalescent process reducing *n *lineages into 1 is obtained by multiplying the (independent) probabilities for each coalescence event,

(2) $$f(T|N(t))=\prod_{i=2}^{n}\frac{\left(\begin{array}{c} i\\ 2 \end{array}\right)}{N(t_{i})}\exp\left(-\int_{t_{i+1}}^{t_{i}}\frac{\left(\begin{array}{c} i\\ 2 \end{array}\right)}{N(t)}dt\right).$$

One nice feature of coalescent theory is that demographic inference depends only on coalescent times and so can be coupled with any method which can estimate the genealogy in a statistically consistent manner. There are numerous examples of the coalescent theory being coupled with different data and estimation procedures for the coalescent times. Tavare *et al *estimated coalescent times from genetic data using only the number of segregating sites while assuming a known demographic function [10]. Coalescent theory has also been used within Markov chain Monte Carlo (MCMC) algorithms to estimate constant [11] and exponentially growing [12] population sizes using microsatellite data. More recently the Skyline Plot, a maximum likelihood estimator (MLE) of the demographic function for a known genealogy, was introduced [13]. This was further extended in the form of the Bayesian Skyline Plot (BSP) [14], a MCMC method that estimates the demographic function directly from sequence data and provides much needed credible intervals. This paper extends the BSP further by allowing the joint analysis of multiple loci and eliminating the requirement to pre-specify the model dimensionality. An alternative approach using reversible jump MCMC to dynamically change the dimensionality of the demographic model was previously introduced [15], but was implemented for a known genealogy only. More recently an alternative which uses Gaussian Markov random fields (GMRFs) to achieve temporal smoothing of the population sizes has been developed [16], but this method does not currently support multiple loci.

### Inferring population size from coalescent times

Estimating changes in population size is a challenging task, especially when the magnitude of the change is small. Even if the exact coalescence times are known the space of demographic functions capable of generating them is large. In general the coalescent times are unknown and have to be estimated by phylogenetic reconstruction, thus increasing the set of plausible demographic functions further. In addition, the "observation window" is limited by the last coalescent which is on average 2*N *generations in the past. Moreover, under a constant population size it is expected that half of the time to the root of the tree is spanned by just one coalescent interval (one data point), so no dynamics can be inferred, just the average population size over that time span. Similarly (under constant size expectations) just two data points account for 2/3 of the tree, and so on. Thirdly evolutionary bottlenecks (periods with smaller population sizes) have shorter coalescence times, reducing the observation window further.

Faced with these problems, one might be tempted to increase the number of samples. However the returns from such an investment diminish quickly – the additional coalescent events occur inside a small stretch of time. It is much better to add sequences from independent loci from the same population since all loci share the same demographic history. The assumption of independence requires loci from different chromosomes or sufficiently distant to one another to be considered unlinked by recombination. Doubling the number of (independent) loci doubles the amount of information over the whole "observation window". In this paper we introduce the Extended Bayesian Skyline Plot (EBSP), a new variable-dimension Bayesian method for inferring non-parametric population size changes through time from multiple loci. The EBSP builds on the Bayesian Skyline Plot (BSP) [14] in several ways:

• Permits analysis of multiple loci. Any number of unlinked nuclear or mitochondrial loci from individuals in the population may be combined to infer the shared population size history. Each loci may have its own *population factor *which takes care of differences in ploidy and inheritance. For example, In many animals the population of alleles of a nuclear gene is four times greater than that of mtDNA since there are two copies of the nuclear gene in each individual and mtDNA is inherited exclusively maternally in most species. In that case the population factor can be set to 4 for nuclear genes and 1 for mtDNA if inference of mtDNA gene population size is desired, or to 2 for nuclear genes and 1/2 for mtDNA to infer the number of individual animals.

• Uses Bayesian stochastic variable selection. The original BSP required the researcher to arbitrarily choose *i *the number of population size steps, or control points. The demographic function is then constrained to be a piecewise constant function with exactly *i *distinct levels. It is not obvious how to *a priori *choose *i*, and a poor choice may lead to larger credible bounds and in more extreme cases inhibit convergence. The EBSP, in a true Bayesian spirit, lets the data select the appropriate smoothness of the demographic function using Bayesian stochastic variable selection (BSVS) [17-19].

• Supports piecewise linear demographic functions. Because real life population size dynamics tend to be continuous, a piecewise linear demographic function will generally be a more appropriate model than the piecewise constant function used by the BSP.

## Methods

Consider the most simple case of two lineages and constant population size *N*. Given that the time to coalesce was *t*, what can be said about *N*? The maximum likelihood estimate (i.e. the value which maximizes *f*(*t*|*N*)) is *N *= *t *[20]. While this is the best point estimate possible, the large variability inherent in a stochastic process driven by an exponential distribution makes a point estimate unsatisfactory. The Bayesian framework provides a way to quantify the uncertainty using Bayes rule, $f(N|t)=\frac{f(t|N)f(N)}{f(t)}$.

With the natural non informative prior for a scale variable *f*(*N*) = 1/*N *[21] the cumulative probability can be solved which enables us to obtain an explicit numerical solution for the credible interval. This turns out to be [0.093t, 19.504t], an order of magnitude at both sides of *t*. Point estimates from this distribution can also be computed. The mode is $\sqrt{t/2}$, median is *t*/ln(2) and the mean is infinite.

On the other hand, a genealogy with *n *lineages contains *n *- 1 independent observations via the time intervals between subsequent coalescence events, providing a much better estimate of *N*. However, it should be noted that point estimates from a Bayesian inference typically contain a built-in bias. For example the bias for the median in the two lineages case above is on average 44% (1/ln(2) - 1). The bias in real life data sets which contain multiple loci from several individual is naturally much reduced.

### The likelihood of genealogies from multiple loci

The likelihood of the EBSP is derived from *m *genealogies in the form of rooted trees, denoted *G *= {*g*_1_, *g*_2_...*g*_*m*_}, were *g*_*k *_is estimated from *n*_*k *_contemporaneous sequences. The time scale for all genealogies should match that of the target population being estimated, but the substitution rate may vary among loci. For example, mtDNA is known to evolve at a much faster rate than nuclear DNA [22,23] so when combining both in one analysis the difference in substitution rate needs to be estimated. We designate the set of substitution rate parameters *μ *to keep the notation from becoming overly confusing. In addition let *P *= *p*_1_, *p*_2_,...,*p*_*k *_be the population size factor of *g*_*k*_, which accounts for any differences in ploidy and/or mode of inheritance among loci. The internal nodes of each genealogy *g*_*k *_define *n*_*k *_- 1 coalescent event times $u_{k}=u_{k,n_{k}},u_{k,n_{k}-1},...,u_{k,2}$, where *u*_*k*,*j *_is the time *j *lineages have coalesced into *j *- 1. The start point is fixed at zero, $u_{k,n_{k}+1}=0$.

**Figure 1:**
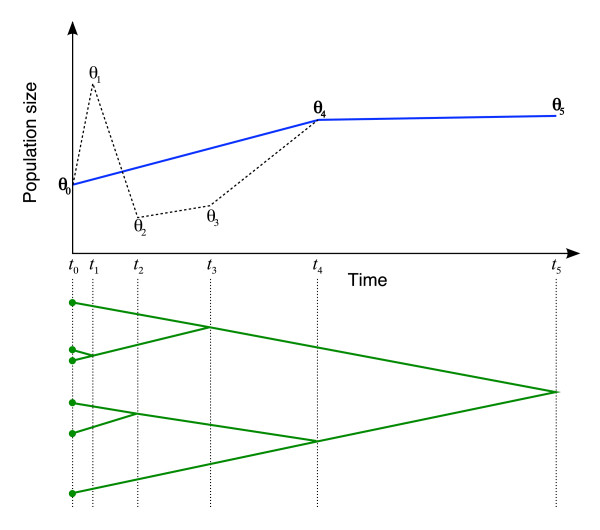
**Constructing a demographic function from population values and indicators**. The demographic function (solid line) is a piecewise linear function whose X axis points (time) are a subset of the set of all coalescent times, and whose Y axis values (population size) are the value of the population parameter (*θ*) at this point. Time starts at zero (the present) increases as we move further into the past. The indicator parameter (*λ*) determines the subset to use for the X axis. In the instance shown above only three of the six population values are used. *λ*_4 _= *λ*_5 _= 1, *λ*_1 _= *λ*_2 _= *λ*_3 _= 0.

Now let *T *= {*t*_0 _= 0, *t*_1_, *t*_2_,...,*t*_*n*_} ∈ ∪_*k *_*u*_*k *_be the vector containing all $n=\sum_{k}\left(n_{k}-1\right)$ coalescence times in sorted order. The demographic function is defined by the population size and *indicator *vectors, Θ and Λ. Θ = {*θ*_0_, *θ*_1_,...,*θ*_*n*_} where *θ*_*j *_is the effective population size at time *t*_*j*_, and Λ = {*λ*_0 _= 1, *λ*_1_, *λ*_2_,...,*λ*_*n*_}, *λ*_*j *_∈ {0, 1}. Θ values whose indicator is *off *(zero) are inactive and do not contribute to the demographic function. One such construction is demonstrated in Figure 1.

Let $r=\sum_{j=1}^{n}\lambda_{j}$ be the total number of active indicators and define the list of active population size parameters $\hat{\Theta }={\hat{\theta }}_{0},{\hat{\theta }}_{1},...,{\hat{\theta }}_{r}$,

$${\hat{\theta }}_{j}=\{\begin{array}{l} \begin{array}{cc} \theta_{0} & \text{if }j=0 \end{array}\\ \begin{array}{cccc} \theta_{m} & \text{if }\lambda_{m}=1 & \text{and} & j=\sum_{i=1}^{m}\lambda_{i} \end{array} \end{array}.$$

The corresponding times as defined in a similar way,

$${\hat{t}}_{j}=\{\begin{array}{l} \begin{array}{cc} t_{0} & \text{if }j=0 \end{array}\\ \begin{array}{cccc} t_{m} & \text{if }\lambda_{m}=1 & \text{and} & j=\sum_{i=1}^{m}\lambda_{i} \end{array} \end{array}.$$

Now it is possible to define the resulting piecewise linear demographic function,

(3) $$\theta (t)=\{\begin{array}{ll} {\hat{\theta }}_{j}+({\hat{\theta }}_{j+1}-{\hat{\theta }}_{j})\frac{t-{\hat{t}}_{j}}{{\hat{t}}_{j+1}-{\hat{t}}_{j}} & \text{if }{\hat{t}}_{j}\leq t\leq {\hat{t}}_{j+1}\\ {\hat{\theta }}_{r} & \text{if }r>{\hat{t}}_{r} \end{array}.$$

The per-locus demographic function depends on the population size factor as well, ${\hat{\theta }}_{k}(t)=p_{k}\theta (t)$.

The use of the ranked coalescence times in the construction of the demographic function is natural since from equation 1 each interval contains the same amount of information. Associating one parameter with each "coalescent interval" (i.e. an interval where all lineages survive terminated by a coalescent event) helps to avoid over specifying the demographic function. This approach has been used in the BSP [14] and more recently in the Bayesian skyride method [16].

The likelihood calculation requires integrating ${\hat{\theta }}_{k}{\left(t\right)}^{-1}$ over one or more intervals $\left[{\hat{t}}_{j},{\hat{t}}_{j+1}\right]$. By definition the time points $t_{i}\in [{\hat{t}}_{j},{\hat{t}}_{j+1}]$ partition the interval so that the demographic function is linear on each sub interval. The integral over the interval is then obtained by summing over those sub intervals using the easily derived integral of the inverse of *y*(*x*) = *ax *+ *b *over an arbitrary interval [*v*_0_, *v*_1_],

(4) $$\int_{v_{0}}^{v_{1}}\frac{1}{y(x)}dx=(v_{1}-v_{0})\frac{\ln(\frac{y(v_{1})}{y(v_{0})})}{y(v_{1})-y(v_{0})}.$$

The log likelihood of each genealogy is

(5) $$lnf_{G}(g_{k}|\Theta ,\Lambda )=\sum_{i=n_{k}}^{2}ln\frac{\left(\begin{array}{c} i\\ 2 \end{array}\right)}{{\hat{\theta }}_{k}(u_{k,i})}+\int_{u_{k,i+1}}^{u_{k,i}}\frac{\left(\begin{array}{c} i\\ 2 \end{array}\right)}{{\hat{\theta }}_{k}(t)}dt.$$

The prior of Θ is composed from individual priors on *θ*_*j*_, where each value is drawn from an exponential distribution with a mean of *ϕ*.

(6) $$f_{\Theta }(\Theta ,\phi )=f_{\phi }(\phi )\prod_{j=1}^{n}\frac{1}{\phi }e^{-\theta_{j}/\phi }.$$

The priors on all of the *θ*'s contribute to the posterior, but only active ones participate in the demographic function, and therefore the coalescent likelihood in equation 5. Therefore, when inactive, *θ*_*j *_follows just the prior distribution but when active it follows the posterior (prior and coalescent).

When an appropriate prior value for the mean population size is not known in advance *ϕ *may be estimated in a hierarchical manner. A suitable prior distribution is selected and *ϕ *is allowed to change under that prior. One may choose the scale-free reference prior (*f*_*ϕ*_(*ϕ*) = 1/*ϕ*) as the least informative option or a so-called "diffuse" prior such as a log-normal with high variance. Note that using the reference prior may lead to very slow mixing and so it may be advisable to follow a "empirical Bayes" approach as suggested in [15] and obtain an estimate from the data itself. It is important to remember that since *ϕ *is the mean of an exponential distribution the choice of prior will make little difference unless the amount of data is small. However selecting a *ϕ *(or a prior for it) which is smaller by two orders of magnitude or more than the truth may, in our experience, cause non convergence of the chain (data not shown). Selecting higher values may slow mixing but do not appear to impact convergence. In practice fixing *ϕ *to a large enough value works very well (simulation results not given here).

Our simulation studies used a log-normal prior with a standard deviation in log-space of 2 and the mean (in real space) was randomly selected uniformly from the interval [0.5$\hat{\phi }$, 3.5$\hat{\phi }$] ($\hat{\phi }$ is the mean of the true demographic function averaged over the ages of the simulated trees).

The prior on Λ is chosen as if *r *= ∑_*i*_*λ*_*i *_is drawn from a (truncated) Poisson distribution with a mean of $\bar{\lambda }$ = ln(2), then uniformly from all binary vectors containing exactly *r *ones.

(7) $$f_{\Lambda }(\Lambda )\propto {\left(\begin{array}{c} n\\ r \end{array}\right)}^{-1}\frac{e^{-\bar{\lambda }}{\bar{\lambda }}^{r}}{r!}.$$

The choice of $\bar{\lambda }$ = ln(2) gives a 50% prior weight to a constant population size and 50% to a non-constant one. This prior is used in all simulations and analyses unless specifically noted. The prior parameter $\bar{\lambda }$ may be increased to indicated stronger prior belief in a non-constant demographic. However data with some support for changes in population size will tend to overcome the prior and a prior that focuses probability on a small number of change-points will tend to result in narrower credible intervals.

The method works for serially sampled data as well, and has been implemented in the software package BEAST [24] for both contemporaneous and time-stamped data. The definitions above can be easily modified to accommodate genealogies containing non-zero tip times but the notation is even more cumbersome and therefore for clarity is not given here. For an example of BEAST XML input file of 32 loci [see Additional file 1].

### MCMC Implementation

The posterior distribution to be sampled for the EBSP is

$$\begin{array}{r} f(\Theta ,\Lambda ,G,\phi ,\mu |D,P)=\frac{1}{Z}\left[\prod_{k=1}^{m}f_{D}\{D_{k}|g_{k},\mu \}f_{G}(g_{k}|\Theta ,\Lambda )\right]\\ f_{\Theta }(\Theta ,\phi )f_{\Lambda }(\Lambda )f_{\mu }(\mu ). \end{array}$$

The term *f*_*D*_{*D*_*k *_| *g*_*k*_, *μ*} is the genealogy likelihood calculated from the data and model parameters using standard methods [25]. The genealogy can be sampled by any of the published methods available in BEAST [24].

Inactive *θ*'s can be drawn directly from the prior *θ*_*i *_~ *Exp*(*ϕ*). The posterior distribution for active *θ*'s has no closed form and is sampled by applying generic scale-parameter proposal schemes (the scale operator) available in BEAST to each parameter of population size.

The indicators are sampled by combination of a *bit-flip *operator and Poisson weighting. By itself the bit-flip generates samples with a stationary probability of $Pr(\Lambda )=\frac{1}{(n+1)\left(\begin{array}{c} n\\ r \end{array}\right)}$ taking care of the first term in equation 7, while the Poisson weighting accounts for the second half.

The bit-flip operator uniformly picks one bit of Λ and flips it. Since the transition probability for the move and the reverse move are equal to 1/*n *the Hastings ratio [26] for changing a 1 to a 0 is

$$\frac{\pi (\lambda )q(\lambda^{\prime }|\lambda )}{\pi (\lambda^{\prime })q(\lambda |\lambda^{\prime })}=\frac{\pi (\lambda )}{\pi (\lambda^{\prime })}=\frac{\left(\begin{array}{c} n\\ r \end{array}\right)}{\left(\begin{array}{c} n\\ r-1 \end{array}\right)}=\frac{n-r+1}{r}.$$

The Poisson weighting contributes an additional factor of r/$\bar{\lambda }$.

The ratio for changing a 0 to 1 is derived in the same way and is equal to $\frac{r+1}{n-r}\frac{\bar{\lambda }}{r+1}$.

## Results and Discussion

To demonstrate some of the features of EBSP and the inference of demographic functions in general, this section describes the results of simulations performed using an implementation of EBSP in BEAST [24] (The Beast-EBSP program is available from http://beast-mcmc.googlecode.com/ and an example XML input file is available as an additional file). Simulations are invaluable during the development of a new method and in addition may provide insight into the properties of the EBSP in particular and demographic inference in general. Having said this, the simulation results we present here are aimed to serve only as examples of the various issues that need to be considered, since only a small subset of parameter combinations can be feasibly explored.

Here are the steps taken to generate a simulated data set. First pick a demographic *N*(*t*), number of loci, ploidy and number of samples for each loci. Then, for each locus, a genealogical tree *T *is simulated under *N*(*t*) and the coalescent process. Then a set of sequences is simulated using *T *and a DNA substitution model (which has its own set of parameters). The sequences are then used as the raw data for an MCMC run, and posterior samples from the run are used to estimate the population size history (and other parameters of the model if required). Note that while the simulated data contains the complete set of loci for each "individual" this is not strictly necessary. Data sets containing only a subset of loci for some individuals can be used as well.

### Bayesian summary for functions

Summary statistics computed from posterior samples are the standard and straightforward way to present results of an MCMC run. When dealing with functions, however, there is no direct equivalent for single value statistics such as a median or Highest/Central Posterior Density (HPD/CPD [27]). For example there is no obvious way to pick one population size function out of the MCMC sample which represents a "middle" or a "center" in the same way a median does for single value statistics. However such statistics are highly useful for the purpose of visualization, quantification and comparison and are easily constructed from multiple estimates at specific grid points [28].

A piecewise linear function connecting the median population size estimated at specific time points is a natural choice for the median when posterior samples are piecewise linear functions. Since the demographic has a natural resolution limit *n*, the total number of coalescence events, we propose that the construction of the median demographic also use *n *points which are estimated by the mean times of the ordered coalescent events over all posterior trees. This is different from the approach currently taken by Tracer [29] which uses a fixed (say 100) evenly spaced points. This inappropriately ignores the natural spacing of coalescent events and lumps together information from several intervals at the beginning while half the points at past times are essentially based on the age of a single coalescent interval. It is of course possible to refine the time axis further by introducing more points between mean coalescent event but this adds only a small amount of information and we prefer to keep a visual clue about of the amount of data the estimate is based upon. It should be noted that these choices are relevant only for summarizing the posterior distribution. Specific hypothesis tests related to population size history should always be constructed directly from the posterior samples and not from any summary statistic.

**Figure 2:**
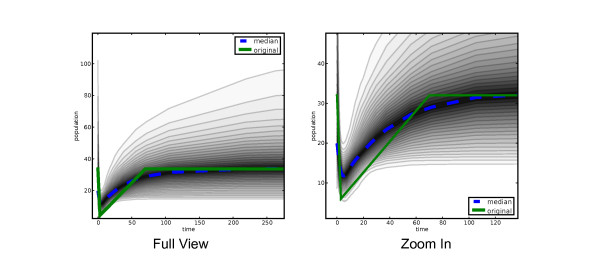
**Posterior distribution of the recovered demographic function**. The recovered demographic from a Bayesian analysis can be presented using gray-scale shading to represent CPD levels (quantiles). The dotted line shows the median. The solid line represents the "truth", i.e. the demographic function used to generate the simulated data.

The same procedure is used to build the mean, Central or Highest Posterior Density functions through time. Figure 2 shows two snapshots from an interactive exploration of one recovered population size history. The figures depict a nested set of credible intervals. We shall denote the *p*-percentile of the posterior density of *N*(*t*) at time *t *as *N'*_*p*_(*t*).

By comparing the median demographic ${N^{\prime }}_{50}(t)$ to the true demographic *N*(*t*) using a function norm, from *t *= 0 to the median root height, *t *= *τ*, we can define a relative recovery error:

$err(N^{\prime }(t),N(t))=||\frac{{N^{\prime }}_{50}(t)}{N(t)}-1|{|}_{2}=\sqrt{\int_{0}^{\tau }{\left(\frac{{N^{\prime }}_{50}(t)-N(t)}{N(t)}\right)}^{2}dt}.$

Note that while the word *error *above is used to describe the average distance between an estimate and the truth, this quantity contains a bias component which is to be expected of a Bayesian estimate of a scale parameter.

The size of the relative 95% credible interval is defined in a similar way:

$$size(N^{\prime }(t),N(t))=||\frac{{N^{\prime }}_{97.5}(t)-{N^{\prime }}_{2.5}(t)}{N(t)}|{|}_{2}.$$

Another useful statistic is the frequentist coverage of the estimator, which measures the percentage of time the true value of the demographic was inside the 95% credible interval:

$$cover(N^{\prime }(t),N(t))=\frac{1}{\tau }\int_{0}^{\tau }I({N^{\prime }}_{2.5}(t)\leq N(t)\leq {N^{\prime }}_{97.5}(t))dt,$$

where *I *is the indicator function.

### Estimation when population size is constant

The first set of simulations was set up to reflect a typical data set with current technology. We simulated three nuclear markers and one mtDNA gene from 12 individuals sampled from a constant-sized population of 50,000. Twenty-four recombination free sequences of length 1600 were generated for each nuclear marker but only 12 for the mtDNA, mimicking a situation where both alleles are sequenced from each nuclear locus. We used the HKY85 [30] substitution model to describe mutation within each locus with *κ *= 2.5 for the nuclear loci and *κ *= 15 for the mtDNA. The mtDNA locus substitution rate was set to 10^-7^, 20 times faster than that of the nuclear (5 × 10^-9^), producing a mtDNA tree with an average height of approximately 0.01 substitutions (2 × 5 × 10^4 ^× 10^-7^). Note that although the MCMC runs try to mimic real life usage and estimate all unknown parameters (*κ*, mtDNA *μ *etc), only results for the demographic function are given below.

In this first set of simulations 100 independent data sets were generated and each was analyzed in three different ways: (a) with the EBSP, (b) under the assumption of a constant size coalescent model, and (c) under the assumption of a constant population size with the genealogy (i.e. coalescent times) fixed to the simulated values. This allows us to measure the relative contributions to the overall uncertainty in the demographic function estimate that arise from (i) model averaging, (ii) uncertainty in coalescent times and (iii) the intrinsic stochastic nature of the coalescent process. This approach is quite useful and is used again later. The results are summarized in Table 1.

**Table 1:** Constant population size, N = 50,000

model	% inside 95 HPD	median relative error	median relative bias	HPD relative bounds
EBSP	97.65%	0.22	0.053	1.23

constant population size	96%	0.17	0.009	0.9

constant population size, fixed trees	96%	0.086	0.006	0.44

This initial set of simulations demonstrates that even with four loci the credible interval has the same order of magnitude as the population size itself. Furthermore, the uncertainty that arises from estimating the coalescent times from the genetic data (about 40%) is comparable to the uncertainty arising from estimating the population size from the coalescent times. The remaining 20% is due to using the EBSP when the demographic function was in fact constant (this can be considered to be the price of model averaging).

Using longer sequences improves the estimates of coalescent times, and thus reduces that component of the uncertainty. This is demonstrated by a second set of simulations which use the same model parameters but with sequences of length 16000 bp. The results are shown in Table 2.

**Table 2:** Constant population size, 10× more sites

model	% inside 95 HPD	median relative error	median relative bias	HPD relative bounds
EBSP (16000 bp)	96.84%	0.13	0.034	0.77

Longer branch lengths, either through increased mutation rate or increased population size would also lead to less uncertainty in the estimates, so that short sequences would be sufficient provided the product of population size and mutation rate is much larger than in our first simulations. This is demonstrated by the results from 100 MCMC runs in which the population is ten times larger in size (Table 3).

**Table 3:** Constant population size, 10× population size (N = 500,000)

model	% inside 95 HPD	median relative error	median relative bias	HPD relative bounds
EBSP	98.94%	0.12	-0.008	0.71

constant population size	99%	0.1	-0.024	0.54

constant population size, fixed trees	99%	0.079	-0.005	0.44

The second and third sets of simulations show how longer sequences or a larger population effectively narrow the credible intervals by allowing better estimation of coalescent times. When studying smaller populations it is advisable to use longer sequences in order to ensure accurate estimates of the branch lengths. However we note that this is not necessarily possible with nuclear loci that experience large amounts of recombination.

### Number of loci vs. error

**Figure 3:**
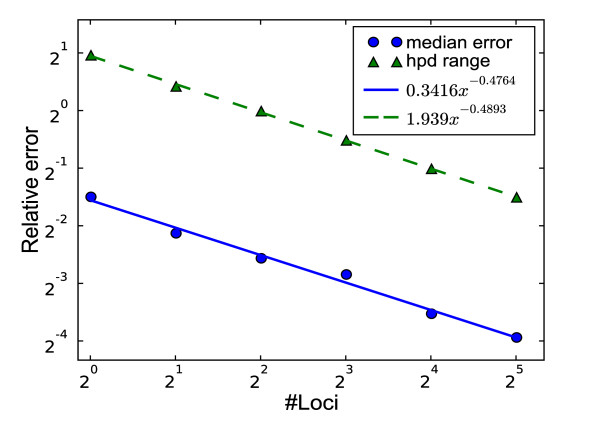
**Error in demographic recovery as a function of number of loci**. The error (on log-log scale) reduces linearly as the number of loci increases (solid line). Using 4 times the number of loci halves the error. Likewise, the 95% HPD interval also reduces linearly on a log-log scale with increasing loci (dashed line), and also appears to reduce by a factor of 2 with a quadrupling of the number of loci.

While increasing sequence length improves the estimation of coalescent times, only additional loci can reduce the variability in estimating the population size function from those coalescent times. The next set of runs show the effect of increasing the number of loci. A single locus was simulated for 5 individuals, then a second one was added to make two, then two more to make four, doubling up to 32. For this analysis the simulated coalescent times were used directly, so that we could focus exclusively on the contribution of the variability in the coalescent process to the 95% HPD interval. The results of 100 such runs are shown in Figure 3.

Empirically, both the error and 95% credible interval are reduced by a factor of $\sqrt{2}$ when doubling the number of loci. This suggests that the relation between median error/HPD interval size and the number of coalescent points (*sample size*) follows the pattern of simpler cases where doubling the sample size reduces the variance by half. A rigorous proof requires an analytical solution for the median and HPD and is a non trivial task beyond the scope of this paper.

### Number of samples vs. error

**Figure 4:**
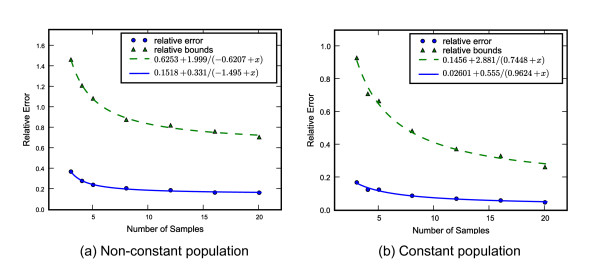
**Error in demographic recovery as a function of number of samples**. Error and credible intervals are shown as a function of the number of sampled individuals per loci for 16 independent loci. Both the error (solid line) and the HPD interval (dashed line) reduce with increasing number of sequences but both converge to a positive limit, that can only be improved on by sampling more loci. A best fitting line of the form $y=a_{0}+\frac{a_{1}}{a_{2}+x}$ is shown and serves only to highlight the general trend.

Increasing the number of sampled individuals per locus also improves the estimates but the effect is much more modest. Figure 4 shows the results of multiple runs with 16 loci but using 3 to 20 individuals. For each case 100 simulations were made with both a constant and a non-constant demographic function. The non-constant population size function linearly decreases from *N *to *N*/4 back in time on the interval [0, 0,375*N*] generations, and is constant at *N*/4 at earlier times. Again the raw data were the coalescent times rather than sequence data.

The exact form of the errors is unclear but it does seem to fit an inverse relation with a positive limit. The lower bound depends on the nature of the demographic and on the number of loci as well. Unsurprisingly the bounds and error for the constant demographic are smaller than those for the non-constant case, but the total amount of reduction is approximately the same beyond 8 samples. For example using 16 samples instead of 8 gains an additional reduction of the HPD bounds by 16% and 14% respectively.

### Detecting evolutionary bottlenecks

Evolutionary bottlenecks present a tough challenge for reconstruction of population size history. Periods of low population size increase the rate of coalescence and limits the number lineages that survive the bottleneck, therefore severely reducing the ability to detect changes in population size prior to the bottleneck time. Interest in the effect of evolutionary bottlenecks on genetics predates the coalescent. For example Nei and colleagues investigated the effect of a population bottleneck on the expected heterozygosity for a neutral locus [31]. A more recent study [32] demonstrates the difficulties in analyzing population structure from contemporary sequences using several methods.

**Figure 5:**
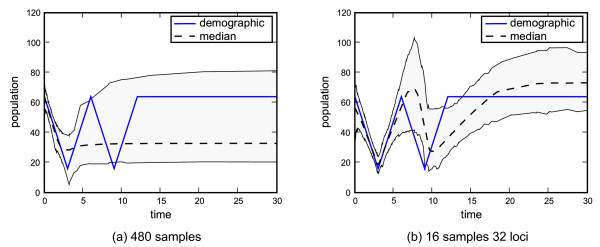
**Multi-locus recovery of a demographic function with evolutionary bottlenecks**. Using multi-locus data enables the detection and accurate recovery of population bottlenecks. The true demographic function is shown as solid line, while the dotted line indicates the posterior median.

Given enough data the EBSP can detect past population bottlenecks. Figure 5 shows the result of running one locus from 480 individuals compared with 32 loci from 16 individuals for an identical demographic containing two bottlenecks in quick succession.

While there is an equal number of coalescent events in both cases, only the analysis of multiple loci is able to "see" past the first bottleneck and even past the second. It is important to stress that this is a carefully constructed example. Lowering the population size at the bottlenecks or changing the difference between the maximum and minimum population sizes will alter the number of loci required for a successful recovery.

### Testing for a non constant demographic function

**Figure 6:**
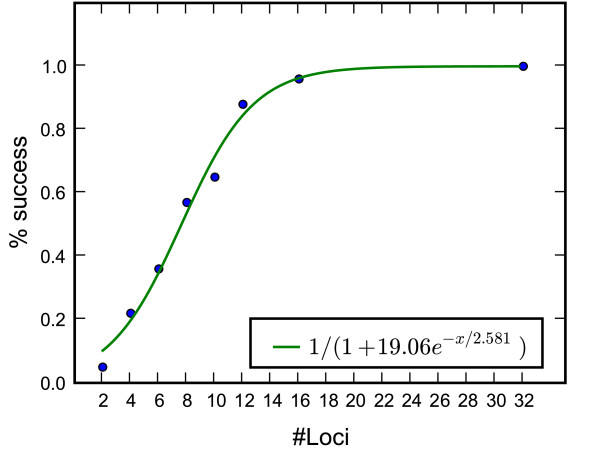
**Testing statistically for the presence of demographic changes**. The success rate of rejecting a constant demographic as a function of number of loci.

The EBSP lets the data itself select the correct dimensionality of the demographic function and so allows statistical inferences regarding the number of change points in the population size function *N*(*t*). This example examines the relation between the amount of data and the frequentist coverage for the most simple case involving a single change from a constant population size to linear growth. Going back in time, the target demographic is linearly decreasing from *N *to *N*/4 on [0, 3/8*N*] generations, and remains constant at earlier times. A hundred data sets were simulated for different numbers of loci between 2 and 32, each sampled from 8 individuals. For each data set the posterior estimate and credible set of the number of control points in the demographic function was recorded. The true demographic function had one change in population size at 3/8*N *time ago. Therefore, runs in which the HPD of the number of change points excluded 0 but contained 1 were recorded as a success since they rejected the null hypothesis of a constant population size. Runs in which the HPD contained 0 were counted as a miss. No run failed to include 1 change in the credible interval. The mean success rate as a function of numbers of loci is shown in Figure 6. With 16 loci the success rate was greater than 95%, while it is quite low at around 20% for 4 loci. It seems to follows a logistic-like shape, but the small number of points does not allow us to elucidate the relationship more precisely.

### Effect of DNA sequencing errors

DNA sequencing typically involves several stages and each may introduce errors, from replication during PCR [33] to incorrect base calling [34]. Realistically modeling the effects of such errors on sequence data is difficult, but even a simplistic approach may prove instructive in describing their impact on demographic inference. This example reuses the setting and sequence data of the first example in which the population size was constant, but randomly changing bases at some fixed rate. This degraded data is then reanalyzed in the same way as the first experiment. Table 4 shows the results of two runs at 0.01% and 0.1% error rates.

**Table 4:** The effect of DNA sequencing errors

	% inside 95	median relativeeerror	median relative bias	HPD relative bounds
Original data, no errors HPD	97.65%	0.22	0.053	1.23

0.01% sequencing error	96.05%	0.53	0.29	1.95

0.1% sequencing error	73.97%	29.54	12.22	65.28

For this particular setting an error rate of 0.1% has a catastrophic effect. Even a rate of 0.01% was enough to double the size of the credible intervals. It is clear that data quality is an important prerequisite for a successful population genetic analysis.

### Hepatitis C Virus in Egypt

The epidemic history of Hepatitis C virus in Egypt has been previously analyzed using both the BSP [14] and parametric Bayesian coalescent analysis [35]. We take another look at the data to examine the two non-parametric methods side by side and investigate the effects of prior and model choice.

**Figure 7:**
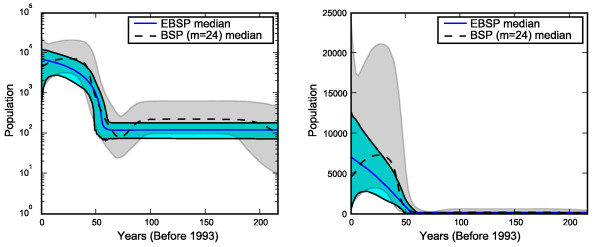
**Hepatitis C virus data: comparison of BSP and EBSP**. Comparison of a EBSP (blue) and BSP (gray) analysis of the Hepatitis C virus data. On the left side the Y axis is in log scale, and linear on the right. Both methods agree on the trend but differ due to model and prior choices. Credible intervals from the EBSP are smaller.

The single locus data consists of an alignment of 63 partial gene sequences of length 411 bp [36]. The results of two MCMC runs, the first using piecewise-constant BSP with *m *= 24 and the second using the piecewise-linear EBSP ($\bar{\lambda }$ = ln(2)) are shown in Figure 7.

Drummond *et al *[14] note that the sequences "contain ample phylogenetic information". Our simulation studies suggest that while 63 samples seem sufficient, a single loci can only detect general trends and not finer details. In this case both methods agree on a sharp decline (going back in time) at around 50–60 years ago. However the BSP favours a constant demographic until that time, while the EBSP favours steady decline and constant demographic from 60 years ago backwards. We suggest this difference is an effect of model and prior choice and we present one run (out of several) which supports this view.

**Figure 8:**
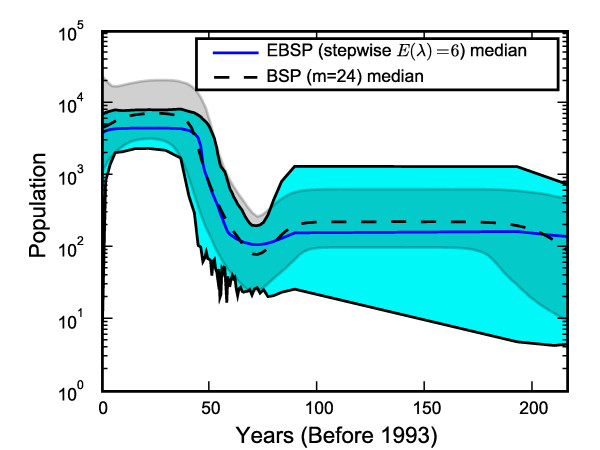
**Hepatitis C: EBSP stepwise model and *E*($\bar{\lambda }$) = 6**. Matching more closely the model and prior between the EBSP and BSP brings the results much closer. Note the negative effect of the prior choice *E*($\bar{\lambda }$) = 6 on the size of credible bounds.

Figure 8 shows the result of running the EBSP using the piecewise constant model and an expected prior average number of change points of 6. It is evident that making the priors more similar reduce the difference in the final estimates. The advantage of using the EBSP for a single loci is that it gives a Bayesian estimate of the number of changes actually present in the data. In this case the credible set was {1, 2} which strongly supports a non constant demographic, but a mean of 1.35 suggests that choosing *m *= 24 for the BSP is too high.

### The population size history of Drosophila ananassae

Aparup *et al *[37] investigated the demography and population structure of *Drosophila ananassae*. The authors collected samples from 160 individuals over 16 geographic locations and sequenced 10 fragments whose length ranges from 371 to 487, giving a total of 650 kb.

**Figure 9:**
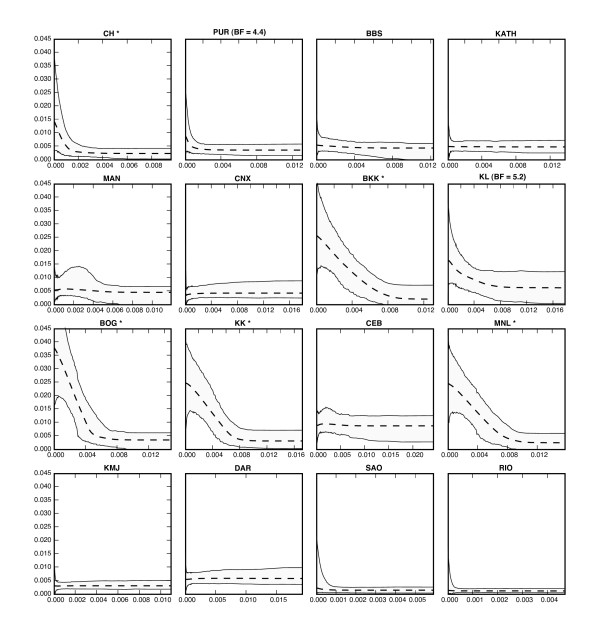
**16 EBSP runs on Drosophila ananassae**. The estimated demographic history for each of the 16 locations. The abbreviated names of the sampling locations are given above each plot. An asterisk (*) is appended to locations whose demographics are non-constant according to the EBSP with 95% posterior probability. Locations with some support (i.e. where the Bayes factor $\frac{p(D|r\neq 0)}{p(D|r=0)}$ is greater than 3) are also indicated (PUR and KL).

The result of an EBSP analysis for each of the 16 locations is shown in Figure 9. While the sequence length per loci is relatively short and there are just 529 SNPs in the data-set, our simulation studies suggest the combined power of 10 loci is likely to provide a good overview of the trends in population size changes through time.

Our simulation study has already demonstrated how posterior probabilities of *r *(the number of change points) can be used to infer demographic change. Aparup *et al *found support for population expansion in 4 of the central populations – BKK, BOG, KL, and MNL (Location names are abbreviated as in the original article). Our analysis finds strong evidence for population expansion in 5 of the 16 populations (BKK, BOG, CH, KK and MNL). In each of the five populations the posterior probability of a non-constant population was greater than 95%, i.e. *Pr*(*r *> 0) > 95%.

As a more lenient alternative one may use the Bayes factor $\frac{Pr(D|r\neq 0)}{Pr(D|r=0)}$ to test for the presence of change [38]. The populations of KL and PUR with Bayes factors of 5.2 and 4.4 respectively show "substantial" support according to the suggested interpretation of Kass and Raftery, but whether those values are large enough is a matter of personal preference.

The expansion in the Indian CH and PUR populations started more recently than the expansion in the central areas. The Puri (PUR) population seem to experience a trend which is somewhere in between that of Chennai (CH) to its South-west which shows clear expansion and Bhubaneswar (BBS) to its North-east having no expansion.

**Figure 10:**
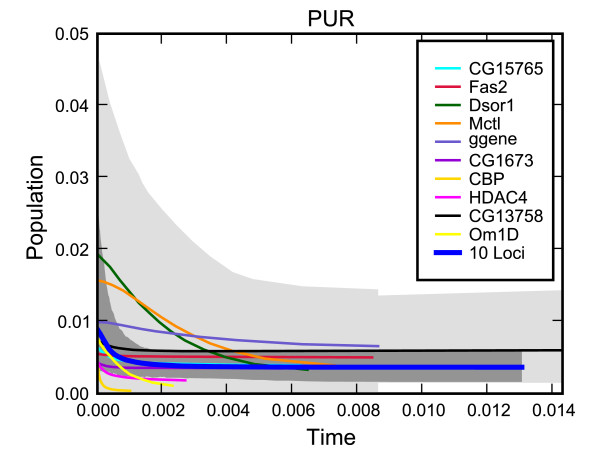
**A detailed look at the Puri Drosophila ananassae population size history**. A detailed view of the Puri (PUR) demographic analyses. The median reconstruction for each of the 10 individual loci is shown, along with the union of the 95% HPD areas in light gray. The thick blue line shows the median for the combined analysis and the 95% HPD is given in darker gray.

The power of multiple loci in improving estimates has already been discussed so we would like to offer here a different visual view of the same effect. We choose the Puri (PUR) area which had the smallest support for population expansion. Figure 10 details the results of running an EBSP analysis for each of the 10 fragments individually and the combined analysis. It is evident a human would find it quite hard to draw conclusions based on the 10 separate runs. The figure also seems to support our conjectured reduction of uncertainty by a factor of about 3 (i.e. about $\sqrt{10}$).

### Performance and Mixing

We would like to briefly comment on the EBSP performance for the two real data sets presented above. The single locus Hepatitis C data was analyzed using both the EBSP and the BSP. In both cases the chain length was set to 100 m and sampled every 2000 states, leaving 49500 samples after removing 10% burn-in. The EBSP run took 2.6 hours on a Quad core Intel Xeon CPU at 2.66 GHz and the BSP took 3.6 hours. The effective sample size (ESS) for the posterior was 5200 for the EBSP and 630 for the BSP, giving a rate of approximately 2 seconds per EBSP effective sample and 20 seconds for the BSP. A closer look reveals that the component responsible for the slower mixing is the genealogy likelihood, i.e. likelihood of sequence data given the tree (*f*_*D *_{*D*_*k *_| *g*_*k*_, *μ*}). Since the coalescent acts as a prior for the tree it seems that the EBSP is allowing a faster mixing of the coalescent times in the tree by providing a demographic history *N*(*t*) with less variability.

For the *Drosophila ananassae *data set one MCMC chain was produced for each of the 16 locations. Each chain was run 2.75 × 10^7 ^steps and sampled every 5000 steps, leaving 5000 samples after removing 10% burn-in. The ESS for the posterior ranged between 220 and 2957, with a mean of 1154. This wide range may be due to variations between populations in the amount of concordance in estimated population size history among loci. Populations which exhibit conflicting demographic signals among loci are expected to mix slower. However this conjecture is based only on visual inspection of results from the analysis of individuals genes. It is also possible that some improvement might come from allowing variation in mutation rate among loci.

## Conclusion

Multi-locus approaches are becoming more attractive with decreasing DNA sequencing costs and increasing computational power. Felsenstein has recently investigated the ideal combination of samples, sites and loci for a fixed cost under maximum likelihood settings [39]. He found that increasing the number of loci is the most cost effective way of improving accuracy, as well as determining that a small number of samples (around 8) is sufficient. Carling and colleagues find that 25 loci from 10 samples is sufficient to accurately estimate constant population size, again under MLE [40].

The EBSP provides a tool to investigate populations that change size through time, by directly inferring the population size as a function of time using sequence data from multiple loci. The EBSP estimates the dimensionality of the population size function automatically from the data, avoiding model over-specification and its associated noise. The Bayesian nature of the EBSP provide an explicit measure of modeling uncertainty. Since we need less individuals when using multiple loci, computational performance is typically not an issue. Running an EBSP for 10 loci and 10 individuals is approximately the same computational effort as running 1 locus for 90 individuals. Mixing, however, is always an issue with MCMC and is data dependent. The EBSP may take longer to mix when there is very little information in the data or there is some conflicting signal among the loci analyzed.

The power of the multi-locus approach in providing better estimates of population size under the stochastic coalescence process is clearly demonstrated by simulation results. However, the results show that taking care of other factors is important too: quality of sequence data, sufficient number of sites and enough samples all help to provide better estimation of coalescent times. Those improvements are reflected directly in the bias and credible intervals of the demographic function estimation.

The results emphasize the importance of support measures when inferring population size history. The inherent uncertainty in population size inference is still substantial even for the large data-sets being collected nowadays. Therefore, it is important to always provide credible intervals with any estimate of population size. From our investigations it appears that high-quality data sets of 16 unlinked loci or more should be used to accurately infer population size history. A relatively small number of individuals is sufficient, but more individuals still provide tangible benefits for data sets using less loci. We are currently working on an extension of this work to the estimation of a species tree from multiple gene trees.

## Authors' contributions

AJD conceived the idea and supervised the development of the method and software. JH wrote the software code, performed all the simulations and data analysis. Both authors contributed to the writing of the article.
